# Right fronto-insular white matter tracts link cognitive reserve and pain in migraine patients

**DOI:** 10.1186/s10194-016-0593-1

**Published:** 2016-02-01

**Authors:** Marian Gomez-Beldarrain, Isabel Oroz, Begoña Garcia Zapirain, Begoña Fernandez Ruanova, Yolanda Garcia Fernandez, Alberto Cabrera, Ane Anton-Ladislao, Urko Aguirre-Larracoechea, Juan Carlos Garcıa-Monco

**Affiliations:** Service of Neurology Hospital de Galdakao-Usansolo, Galdakao, 48960 Vizcaya, Spain; DeustoTech-LIFE/eVIDA, Universidad de Deusto, Vizcaya, Spain; Research and Innovation Department, Magnetic Resonance Imaging Unit, OSATEK, Vizcaya, Spain; REDISSEC, Health Services Research on Chronic Patients Network Research Unit Hospital de Galdakao-Usansolo, Galdakao, Vizcaya, Spain

**Keywords:** Cognitive reserve, Migraine, Chronic migraine, Fractional anisotropy, Insula, Cingulate gyri, Uncinate fasciculus

## Abstract

**Background:**

Structural white matter abnormalities in pain-modulating, regions are present in migraine. Whether they are associated with pain chronification and with cognitive reserve is unclear.

**Methods:**

Prospective, cohort, six-month study of adult patients with episodic or chronic migraine, and controls. Cognitive reserve, quality of life, impact of pain on daily living, depression and anxiety were assessed. Participants underwent a diffusion-tensor MRI to establish the integrity of white matter tracts of three regions of interest (ROIs) implicated in pain modulation, emotion, cognition and resilience (anterior insula, anterior cingulate gyrus, and uncinate fasciculus).

**Results:**

Fifty-two individuals were enrolled: 19 episodic migraine patients, 18 chronic migraine patients, and 15 controls. The analysis of the fractional anisotropy in the ROIs showed that those patients with the poorest prognosis (i.e., those with chronic migraine despite therapy at six months -long-term chronic migraneurs) had a significantly lower fractional anisotropy in the right ROIs. Participants with higher cognitive reserve also had greater fractional anisotropy in the right anterior insula and both cingulate gyri. Multivariate analysis showed a significant association between cognitive reserve, migraine frequency, and fractional anisotropy in the right-sided regions of interest.

**Conclusions:**

Long-term chronic migraine patients show abnormalities in anterior white matter tracts, particularly of the right hemisphere, involved in pain modulation emotion, cognition and resilience. Robustness in these areas is associated with a higher cognitive reserve, which in turn might result in a lower tendency to migraine chronification.

## Background

Migraine is a disabling condition affecting 12 % of the population worldwide, with 1.4–2.2 % of the population suffering from its chronic form (i.e., headache for 15 or more days per month for at least 3 months) [1].

According to the Burden of Disease Study 2013, migraine was the sixth highest cause of disability worldwide; when medication overuse headache is included in the survey, headache disorders rank third among the causes of disability [2].

Episodic migraine progresses to chronic migraine at a rate of 2.5 % per year with the precise mechanisms being unclear. Risk factors include a high attack rate, medication overuse, obesity, depression, low income, as well as non-modifiable conditions, such as childhood stressing experiences ([3].

Although there is no well-established evidence that migraine or medication overuse headache represent risk factors for cognitive deterioration over time [4], little has been investigated about the role of cognitive function on headache chronification. Yet, it is widely accepted that pain is modulated by cognition. Along these lines, we have demonstrated that orbitofrontal cortex (OFC) dysfunction is present in patients with chronic migraine and medication overuse, and is associated with a poorer outcome at 1 year of follow-up [5]. The OFC is a region implicated broadly in the integration of emotion and cognition, and more specifically in the expectation of negative outcomes. Later, we were able to demonstrate, for the first time, an association between low cognitive reserve (CR) and the development of chronic migraine with medication overuse [6]. The most plausible explanation is that people with higher CR have more stable, compact and efficient brain networks resulting in finer tuning of different functions including pain control.

Cognitive reserve (CR) is a newly introduced concept, which refers to performance differences in cognitive processing that make the person more likely to maintain cognitive functioning in spite of disease or damage [7]. Higher levels of education, occupational complexity, and/or premorbid intelligence are associated with lower levels of cognitive impairment than would be expected from a given brain pathology [8]. This cluster of protective factors has been termed “cognitive reserve” by Stern [9] as opposed to “brain reserve”, which refers to differences in brain structure such as neuronal density. The possible neuronal network involved in CR may be a set of inter-related cognitive processes (arousal, sustained attention, response to novelty, awareness and resilience) with a strongly right hemisphere, fronto-parietal localization [8]. The concept of CR had been applied mainly to dementia but there are different studies about the protective effect of CR on the course and prognosis of other nervous diseases such as EM [10], head trauma [11], or drug abuse [12].

On the other hand, it is also known that chronic pain may induce anatomic changes in brain regions related to pain perception and control, particularly the anterior cingulate gyrus, prefrontal cortex and insula [13]. These structures are also implicated in the capacity of resilience [14]. Their dysfunction in patients with chronic pain, including migraineurs, may contribute to poorer pain modulation and to analgesic addiction behavior [15].

Magnetic Resonance Imaging (MRI) in its different varieties has shown structural, functional and connectivity changes in migraine, and has provided insight into its knowledge [16, 17]. In most of these studies the insula and fronto-insular networks are the regions of interest and consistently show abnormalities [18]. Migraineurs are characterized by heightened and anomalous interictal regional connectivity between networks involved in processing upstream sensory information and those that represent the salience of such stimuli in which the anterior insula is the main structure together with the anterior cingulate cortex, and amygdala [19].

Based on our previous studies in which we detected abnormalities in orbitofrontal functions in patient with chronic migraine [5], and also on the association between migraine chronification and medication overuse with low cognitive reserve [6], in this study we sought to investigate the presence of microstructural white matter abnormalities in specific brain regions of these patients that could connect and explain those findings. For this purpose, diffusion-tensor imaging (DTI) was employed. It represents a diffusion-weighted imaging technique that allows the extraction of the diffusion anisotropy characteristics of specific areas of the brain, providing details on their integrity [20]. The regions specifically targeted for this investigation included structures mainly in the frontal area, involved in pain processing and modulation, emotion, cognition and resilience. They specifically include the insula and its frontal connectivity with the cingulate gyrus and limbic (uncinate) regions. These regions of interest (ROIs) were also chosen because they are areas where chronic pain and cognitive reserve may convey and because other studies have demonstrated their impairment in migraine [13].

To this end, we examined patients with episodic migraine, chronic migraine, and controls. At baseline all the participants underwent a DTI-MRI, and filled out specific questionnaires of CR, quality of life, anxiety and depression. Migraine patients were followed for 6 months so as to identify those with the poorest prognosis, i.e., those with persistent chronic migraine despite appropriate therapy.

## Methods

### Study design, participants and patient selection

This is a prospective study in which participants were required to be between 18 and 50 years old and to have had a diagnosis of definite migraine with or without aura according to the criteria of the 2013 version of the International Headache Society (IHS) criteria [21] for at least 1 year before screening, established by one of the neurologists in the study (M.G.B or J.C.G-M.), who evaluated all patients.

Patients were recruited consecutively over a six-month period in the Department of Neurology of our Hospital serving a population of 300 000 inhabitants. Patients were referred by their primary physicians for headache evaluation and were unselected except for their migraine frequency.

Controls were age- and gender-matched and recruited among individuals who consulted for nonspecific complaints, including dizziness, paresthesias, and unsteadiness. They were not taking any medications. None of them suffered from headaches and all of them had a normal neurologic exam and pertinent (i.e., brain or spinal) MRI.

During the initial visit, appropriate abortive therapy was indicated. Comorbid conditions were recorded. A headache calendar was given for a period of 6 weeks on which patients recorded the frequency (days of pain per month) and characteristics of migraine attacks, as well as abortive medication.

During the second visit, scheduled 6 weeks later, patients’ diaries were revised, allowing for their allocation into chronic (≥15 days per month) or episodic migraine patients, always according to the 2013 version of the International Headache Society (IHS) criteria [21]. Preventive therapy was instituted when needed. Participants filled out a CR questionnaire, a generic quality of life questionnaire (SF-36), the Migraine Disability Assessment test (MIDAS), and the Beck anxiety and depression inventory. Controls completed the same tests except for the MIDAS. A DT-MRI was performed to all participants.

To avoid pain interference with their performance on the tests and MRI, patients were asked to rate their pain. A verbal rating scale, where 0 equals no pain and 10 equals excruciating pain, was used. Migraine patients scoring **>**1 were asked to fill out the questionnaires and MRI on another day, once their headache had subsided. Patients were scheduled for visits 3 and 6 months later, where their headache frequency was checked based on their diaries. For this study, those patients who still had a pain frequency ≥15 days per month at 6 months were labeled long-term chronic migraneurs (LTCM).

This study was approved by the Ethics Committee of our institution, and all participants signed a written consent.

### Description of tests and questionnaires

#### Cognitive reserve

CR was assessed by a specific CR index questionnaire in Spanish [22, 23], which includes demographic data and eight items grouped into three sections: education, working activity and leisure time. The items are parental education, formal education, courses taken, job, languages spoken, reading habits, musical education, and frequency in practicing intellectually challenging activities. Each item is rated using a response scale with three to six categories. The higher the score, the better the CR, with a maximum score of 25. Quartiles were employed to determine the CR normative scoring levels.

##### General quality of life—SF-36 [24]

The SF-36 is a structured, self-reported questionnaire that includes 36 items measuring health status across eight domains. The scoring system generates subscale scores for physical functioning (PF), role limitations due to physical problems (RP), bodily pain (BP), general health perceptions(GH), vitality (VT), social functioning (SF), role limitations due to emotional problems (RE) and mental health (MH). Two summary scores derive from the SF-36: the physical component (PF, RP, BP and GH) and the mental component (VT, SF, RE and MH). The SF-36 scores range from 0 to 100, with a higher score indicating better health status.

##### MIDAS

This questionnaire assesses headache-related disability [25]. Migraine patients answer five questions about the frequency (days) and duration of their headaches in the last 3 months, as well as how often these headaches limited their ability to participate in activities at work, at school, or at home. Scoring goes from 0 to 5 days, meaning no disability, to >21 days, representing severe disability.

##### Anxiety and depression evaluation

We employed a Spanish-validated version (validated by Sanz J et al. Pearson education. S.A, 2011) of the Beck Depression (BDI) and Anxiety Inventories (BAI) [26]. These questionnaires consist of 21 self-administered items about how the patient has been feeling in the last week. Each question has a set of at least four possible choices ranging in intensity. Scoring between 11 and 18 indicates a mild depression, between 18 and 25 moderate depression, and over 30 severe depression. In the BAI, scoring 21 points or less represents a low anxiety level, between 22 and 35 moderate anxiety level, and over 36 severe anxiety.

### Neuroimaging protocols

#### Anatomical MRI

Subjects were scanned using a Philips 3.0-T Achieva system with a 32-channel head coil (Philips Medical Systems, Best, the Netherlands), and underwent an anatomical acquisition, a high-resolution structural T1-weighted 3D volume, using a spoiled gradient recalled sequence (SPGR-3D, TR/TE 7.4/3.4 msec; flip angle 8°; matrix size, 228°×°227; field of view, 250°×°250; number of slices, 301; in plane resolution 1°×°1°×°1 mm; NSA 1; Total acquisition time: 4’58”). These anatomical scans were used to confirm the location of the DTI measurement in respect of conventional brain landmarks.

#### DTI data acquisition

Following the anatomical scan, subjects underwent a DTI sequence consisting of an axial single-shot EPI (echo planar imaging) with SENSE acquisition (reduction factor of 2), which lasted 4’17”; 60 slices, 2 mm s/th with no gap, were collected to cover the entire cerebrum and brainstem of each subject (TR/TE 6819/81 msec). The acquisition matrix was 112°×°112, reconstructed to 128°×°128 with a field-of-view of 224°×°224 mm for a 1.75°×°1.75°×°2 mm in-plane resolution. Diffusion-weighting was applied along 15 spherically-distributed axes with b-value = 800 s/mm2, in addition to less diffusion weighted image.

#### DTI methods

Image analysis was performed using Oxford Centre for Functional MRI of the Brain (FMRIB) Software Library (FSL), version 5.0.5 (http://fsl.fmrib.ox.ac.uk/fsl/fslwiki/) [27]. Binary masks of every subject’s brain size and shape were calculated using the Brain Extraction Tool (BET) [28]. Subsequently, maps of fractional anisotropy (FA) were worked out for each subject using the function FDT (FMRIB’s Diffusion Toolbox) package, with the diffusion tensor being obtained for each voxel. TBSS (Tract-Based Spatial Statistics) [29] was then applied and individual whole-brain FA data was aligned via non-linear registration with the most representative subject’s brain space and then affine-aligned into MNI152 standard space. All the subjects were then registered in a common space, and a mean FA image was generated a thinned to create a mean FA skeleton representing the centres of all common tracts for all the subjects, with a threshold set at FA = 0.20. After individual values of FA had been aligned, they were projected onto the mean skeleton. To display the image results of the whole-brain was used the FSL view option of FSL 5.0.5.

#### Region of Interest (ROIs) selected and analysis

ROIs were selected using JHU White-Matter Tractography Atlas [30] for the cingulate gyrus and uncinate fasciculus. The MNI Structural Atlas was selected for the insula [31]; in this case, only white-matter tracts were selected. The ROIs analyzed in this study were selected for their known participation in pain processing, perception and modulation.

Between-group statistical analyses were carried out in three different steps, in the first iteration we used anatomical regions to test for differences in the areas we hypothesised as most important. In the second iteration we ran a brain-wide analysis to check for minor changes between the groups, and finally with the output of this iteration we drew masks of the regions where contrasts showed statistically significant differences and we executed a ROI-based analysis using those masks.

In addition to being able to adjust the marks from the atlas to the white matter, the brain was also segmented in the FSL, FAST tool (FMRIB Automated Segmentation Tool), whereby the masks correspond perfectly to the white matter.

To analyse the DTI voxelwise on the whole brain (Brain Wide) we ran a permutation based in inference tool for nonparametric statistical thresholding with FSL’s “randomize” function with 5000 permutations*.* Between-group comparisons for FA values were performed using two-sample t tests. The statistical maps of each group comparison were thresholded at *p* < .05 corrected for multiple comparisons at a cluster level using the threshold-free cluster enhancement (TFCE) option [32]. For visual inspection, we used corrected statistical maps (*p* < 0.05, corrected) to visualize and select the study areas within the contrast.

### Statistical analysis

Descriptive statistics of socio-demographic variables and clinical data were calculated using frequencies and percentages, while the outcomes in FA in the different ROIs were calculated by means and standard deviations.

Socio-demographic, clinical characteristics and questionnaires results differences were compared among the studied groups at baseline, 3 and 6 months. The non-parametric Kruskal –Wallis test was used for continuous variables as well as the chi-square test for qualitative data. In addition, we conducted a specific post-hoc test to identify specific differences between groups, developing a relevant paired test for each pair and Bonferroni’s correction.

At 3 months of follow-up no switch of patients between groups occurred. However, at 6 months, 9 chronic migraine patients had reconverted to the episodic group. For this reason, and to avoid the reanalysis of the same patients in a different group, the comparison was performed between the patients who still belonged to the chronic group (here labeled as LTCM) and controls.

In order to gauge differences in FA in the different ROIs across independent variables, (sociodemographic, clinical and questionnaires results), univariate analysis was developed using ANOVA analysis. After that, a MANOVA was performed to assess the ROIs, which included frontal insula, cingulate gyrus and uncinate fasciculus, as dependent variables, taking into account the laterality. The Wilks’ Lambda was reported for each MANOVA and subsequent individual univariate statistics were reported for all brain regions that were significant after Bonferroni Correction.

All effects were deemed statistically significant at *p* < 0.05. All statistical analyses were performed using SAS System, version 9.2 (SAS Institute Inc, Carey, NC, USA).

## Results

Fifty-two individuals were enrolled and included 15 controls, 19 episodic migraine patients, and 18 chronic migraine patients. Nineteen migraine patients were on preventive therapy during the study. None of the patients reported pain on the day of examination.

The socio-demographic, clinical data, the CR stratification, the values for the baseline quality of life questionnaires, and MIDAS scores are described on Table 1. Nineteen patients were on preventive therapy, 14 of them belonging to the chronic migraine group, and the remaining to the episodic migraine group.

**Table 1:** Socio-demographic and clinical data of participants

	N (%)	Chronic migraine	Episodic migraine	Controls	*p*-value
Total	52	18 (34.62)	19 (36.54)	15 (28.85)	
*Socio-demographic and clinical data*					
Age^a^	43.46 (7.65)	43.78 (7.91)	41.37 (7.86)	45.73 (6.78)	0.2209
Sex (Female)	47 (90.38)	14 (77.78)	19 (100)	14 (93.33)	0.0460
Cognitive reserve					0.0944
- ≤11 (Low)	13 (25.00)	9 (50.00)	3 (15.79)	1 (6.67)	
- 12–15 (Low-Medium)	13 (25.00)	4 (22.22)	5 (26.32)	4 (26.67)	
- 16–18 (Medium-High)	15 (28.85)	2 (11.11)	6 (31.58)	7 (46.67)	
- >18 (High)	11 (21.15)	3 (16.67)	5 (26.32)	3 (20.00)	
*Questionnaires*					
Beck					
- Anxiety					0.1321
- 0–21	46 (88.46)	14 (77.78)	17 (89.47)	15 (100)	
- 22–35	3 (5.77)	1 (5.56)	2 (10.53)	0 (0)	
- ≥35	3 (5.77)	3 (16.67)	0 (0)	0 (0)	
- Depression					<0.0001
- 0−10	34 (65.38)	4 (22.22)	15 (78.95)	15 (100)	
- 11–18.7	8 (15.38)	5 (27.78)	3 (15.79)	0 (0)	
- 18.8−25.4	4 (7.69)	4 (22.22)	0 (0)	0 (0)	
- ≥25.5	6 (11.54)	2 (27.78)	1 (5.26)	0 (0)	
SF-36					
- Mental component scale (MCS)					0.0003
- <50	31 (59.62)	16 (88.89)	12 (63.16)	3 (20.00)	
- ≥50	21 (40.38)	2 (11.11)	7 (36.84)	12 (80.00)	
- Physical component scale (PCS)					0.0003
- <50	32 (61.54)	17 (94.44)	11 (57.89)	4 (26.67)	
- ≥50	20 (38.46)	1 (5.56)	8 (42.11)	11 (73.33)	
MIDAS					0.0807
- 0–5	6 (16.22)	1 (5.56)	5 (26.32)	NA	
- 6–10	7 (18.92)	3 (16.67)	4 (21.04)	NA	
- 11–20	7 (18.92)	2 (11.11)	5 (26.32)	NA	
- ≥21	17 (45.95)	12 (66.67)	5 (26.32)	NA	

*N* Frequency, *%* Porcentage, ^a^Results shown as mean (standard deviation)

There were no significant differences in age, or CR between groups. The stratification according to CR showed that half of the participants scored low or low-medium, while the other half showed medium or medium-high values. As expected, depression scores were significantly higher in CM patients as compared to episodic migraine and controls (*p* < 0.0001). Significant between-group differences were also present in the mental and physical component of the SF-36 with the CM patients showing the worst scores, followed by the episodic migraine patients group and by controls. Migraine disability as measured by MIDAS was not significantly different between migraine groups.

**Fig. 1:**
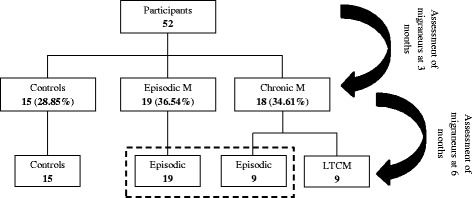
Distribution flow-chart of participants

At 3 months of follow-up, all the 18 chronic migraine patients remained in the CM group, while at 6 months only nine of them still met the CM criteria (i.e., continued with more than 15 days of pain per month) and were labelled LTCM. A participants’ distribution flow-chart is shown on Fig. 1.

The analysis of the FA values in the different ROIs according to socio-demographic, clinical characteristics and CR are shown on Table 2. Analysis at three-month showed that neither the univariate analysis nor MANOVA revealed significant between-group differences in terms of the FA values in the different ROIs.

**Table 2:** Univariate and multivariate analyses of the FA values in the different ROIs according to socio-demographic, clinical characteristics and CR

		ANOVA						MANOVA			
		Anterior Insula		Cingulate gyrus		Uncinate gyrus		Left		Right	
	N	Left	Right	Left	Right	Left	Right	Wilks’ Lambda /F	*p*-value	Wilks’ Lambda/F	*p*-value
		$\overline{x}(sd)$	$\overline{x}(sd)$	$\overline{x}\ (sd)$	$\overline{x}\ (sd)$	$\overline{x}\ (sd)$	$\overline{x}(sd)$				
Total		0.64 (0.05)	0.60 (0.06)	0.75 (0.04)	0.73 (0.05)	0.66 (0.03)	0.68 (0.04)				
Sex					** (0.0322)			0.988/0.19	0.9036	0.873/2.32	0.0870
- Male	5	0.63 (0.05)	0.60 (0.05)	0.75 (0.05)	0.69 (0.04)	0.66 (0.04)	0.66 (0.06)				
- Female	47	0.64 (0.05)	0.60 (0.06)	0.75 (0.04)	0.74 (0.05)	0.67 (0.04)	0.69 (0.03)				
Assessment at baseline and 3 months				*(0.1793)	*(0.0987)			0.924/0.63	0.7068	0.908/0.77	0.5929
- Controls	15	0.65 (0.05)	0.61 (0.05)	0.76 (0.04)	0.75 (0.04)	0.67 (0.03)	0.69 (0.02)				
- Episodics	19	0.64 (0.04)	0.59 (0.07)	0.74 (0.04)	0.74 (0.05)	0.66 (0.03)	0.68 (0.03)				
- Chronic migraneurs	18	0.63 (0.05)	0.59 (0.06)	0.75 (0.05)	0.71 (0.05)	0.66 (0.04)	0.68 (0.05)				
Assessment at 6 months		*(0.0645)	**(0.0457)	**(0.0274)	**(0.0170)	*(0.0645)	**(0.0274)	0.666/3.34	0.0400	0.640/3.75	0.0276
- Controls	15	0.65 (0.05)	0.61 (0.05)	0.76 (0.04)	0.75 (0.04)	0.67 (0.03)	0.69 (0.02)				
- LTCM	9	0.61 (0.05)	0.57 (0.05)	0.72 (0.04)	0.70 (0.04)	0.64 (0.04)	0.65 (0.05)				
Preventive therapy		** (0.0115)	* (0.1261)	* (0.1358)	** (0.0470)	** (0.0420)	* (0.1192)	0.851/2.79	0.0502	0.915/1.49	0.2288
- No	33	0.65 (0.04)	0.61 (0.06)	0.75 (0.04)	0.74 (0.04)	0.67 (0.03)	0.69 (0.03)				
- Yes	19	0.62 (0.05)	0.59 (0.05)	0.74 (0.04)	0.72 (0.05)	0.65 (0.04)	0.67 (0.04)				
Cognitive reserve			** (0.0097)	** (0.0402)	** (0.0202)		* (0.1497)	0.731/1.71	0.0940	0.673/2.20	0.0268
- ≤11	13	0.63 (0.06)	0.59 (0.04)	0.76 (0.04)	0.72 (0.05)	0.66 (0.03)	0.67 (0.04)				
- 12–15	13	0.63 (0.05)	0.56 (0.07)	0.72 (0.05)	0.71 (0.04)	0.66 (0.04)	0.67 (0.04)				
- 16–18	15	0.65 (0.04)	0.63 (0.03)	0.75 (0.03)	0.76 (0.04)	0.67 (0.03)	0.70 (0.03)				
- >18	11	0.65 (0.05)	0.62 (0.06)	0.77 (0.02)	0.74 (0.05)	0.68 (0.04)	0.69 (0.03)				

$\overline{x}\ (sd)$: Mean (standard deviation). *N* Frequency. ** Statistical significance *p* < 0.05. * 0.05 < *p*-value ≤ 0.20. *LTCM* Long-Term Chronic Migraneurs

In contrast, the univariate analysis at 6 months of follow-up revealed that the group of LTCM had significantly lower FA values in the right anterior insula (*p* = 0.0457), left (*p* = 0.0274) and right (*p* = 0.0170) cingulate gyri, and right uncinate fasciculus (*p* = 0.00274) as compared to controls. Specifically, LTCM patients had a lower FA (average 0.04 points) in the right side regions of interest. The multivariate analysis at 6 months dividing the ROIs according to laterality into right (*p* = 0.0276) and left side (*p* = 0.04) confirmed this association.

As shown on Table 2, patients on preventive therapy had lower FA values in the left anterior insula (*p* = 0.0115), right cingulate gyrus (0.0470) and left uncinate fasciculus (*p* = 0.0420). The multivariate analysis showed significant results on left-sided regions.

Regarding CR, participants with higher CR levels had a greater FA in the right anterior insula (*p* = 0.0097) and both cingulate gyri (left, *p* = 0.0402 and right, *p* = 0.0202) according to the univariate analysis. MANOVA showed significant differences for the right-sided ROIs (*p* = 0.0268).

**Fig. 2:**
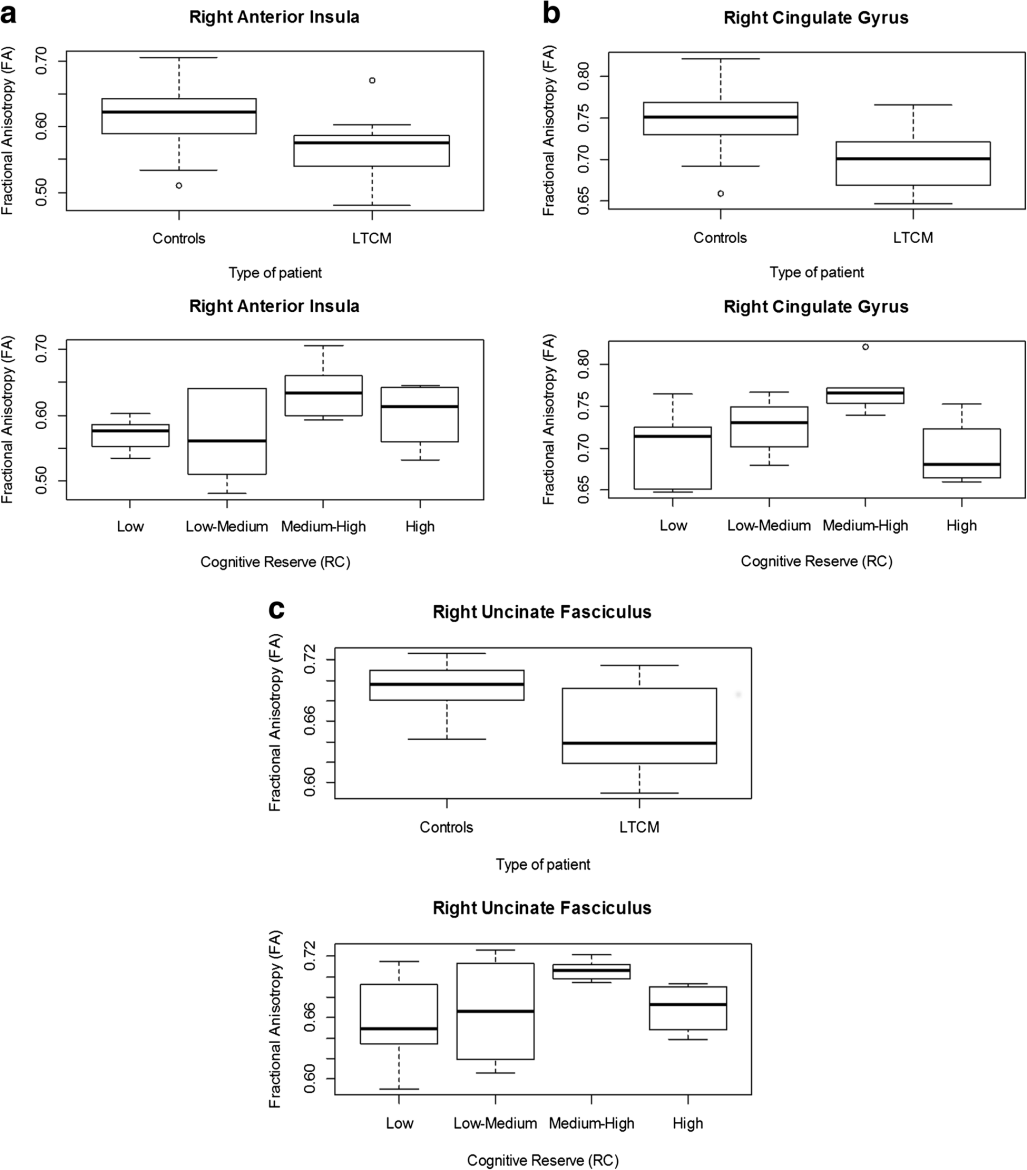
Box-plot showing the FA values of the right-sided ROIs where significant differences were found in LTCM as compared to controls (*top*). The results according to the CR results are also shown (*bottom*)

A box-plot showing the FA values of the right-sided ROIs where significant differences were found in LTCM as compared to controls is depicted on Fig. 2, together with the FA values stratified by CR levels.

**Fig. 3:**
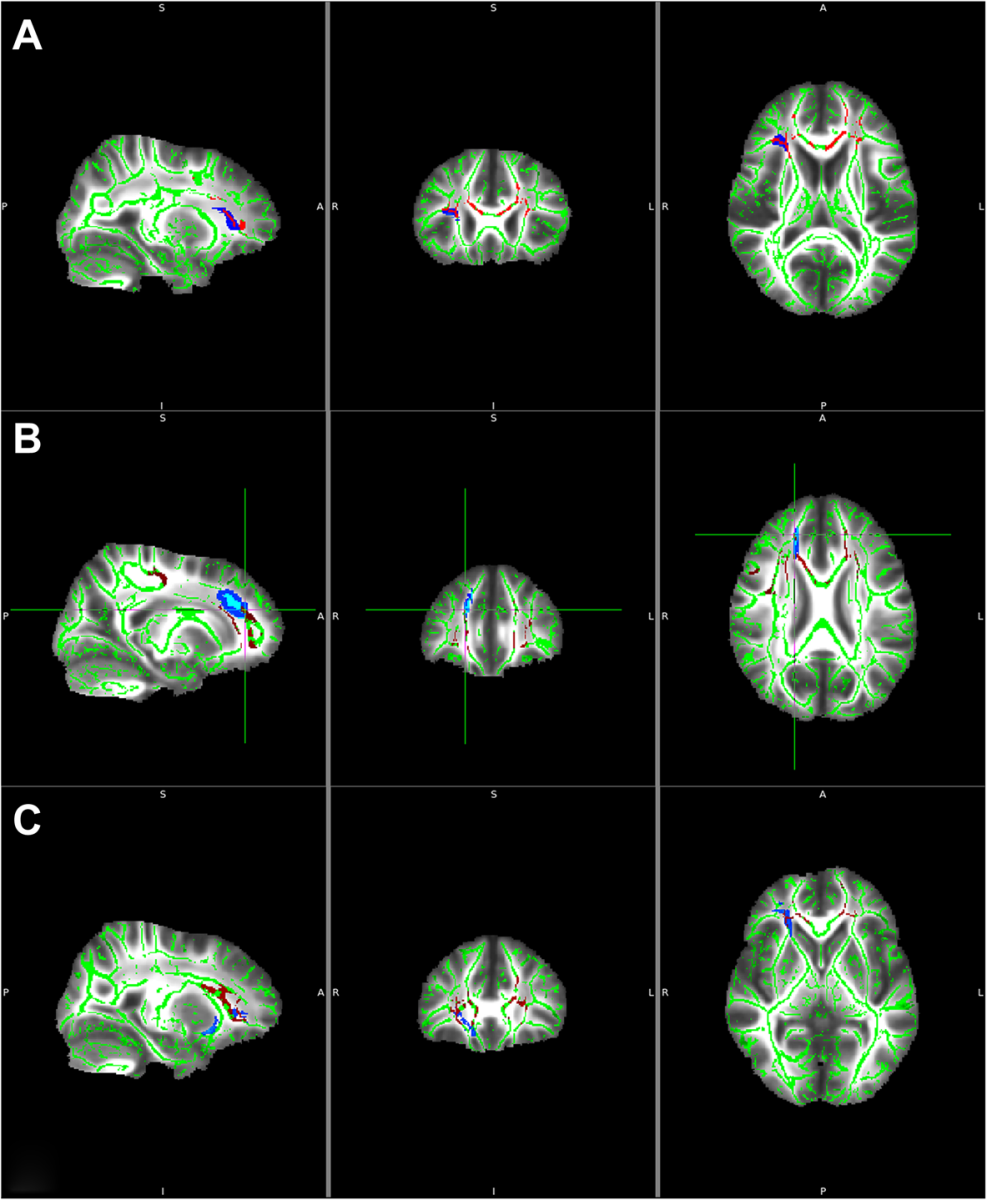
TBSS analysis of FA maps showing the clusters of significantly reduced FA in LTCM patients as compared to the rest of migraneurs in red (TFCE, *p* < 0.05 FWE-corrected). Masks are depicted in blue. **a** right anterior insula; **b** right cingulate gyrus; **c** right uncinate fasciculus. TBSS: Tract-based spatial statistics. LTCM: long-term chronic migraneurs

Figure 3 displays the results of the TBSS analysis of FA maps showing the clusters of significantly reduced FA in LTCM patients in red (TFCE, *p* < 0.05 FWE-corrected). Masks are represented in blue.

Table 3 shows the FA values in the different ROIs according to the stratified scores of the questionnaires. Participants with higher levels of anxiety had reduced FA in the left and right anterior insula (*p* = 0.05) and those with a better SF-36 physical score (Physical Component Score ≥50) had higher FA values in the right (*p* = 0.0173) and left uncinate fasciculi (*p* = 0.0377) as compared to the rest of participants (mean value 0.67, SD 0.04). MANOVA did not reveal significant differences in any of these variables regarding the ROIs laterality.

**Table 3:** Univariate and multivariate analyses of the questionnaires

		ANOVA						MANOVA			
		Anterior Insula		Cingulate gyrus		Uncinate gyrus		Left		Right	
	N	Left	Right	Left	Right	Left	Right	Wilks’ Lambda /F	*p*-value	Wilks’ Lambda /F	*p*-value
		$\overline{x}(sd)$	$\overline{x}(sd)$	$\overline{x}(sd)$	$\overline{x}(sd)$	$\overline{x}(sd)$	$\overline{x}(sd)$				
Total		0.64 (0.05)	0.60 (0.06)	0.75 (0.04)	0.73 (0.05)	0.66 (0.03)	0.68 (0.04)				
Beck											
- Anxiety cat.		* (0.1327)	* (0.0515)			* (0.0996)		0.907 / 1.65	0.1910	0.903 / 1.71	0.1773
- 0–21	46	0.64 (0.05)	0.61 (0.06)	0.75 (0.04)	0.73 (0.05)	0.67 (0.03)	0.68 (0.04)				
- >21	6	0.61 (0.06)	0.55 (0.06)	0.75 (0.05)	0.71 (0.04)	0.64 (0.03)	0.67 (0.03)				
- Depression			* (0.1372)					0.905 / 0.52	0.8576	0.764 / 1.46	0.1723
- 0–10	34	0.64 (0.04)	0.60 (0.05)	0.75 (0.04)	0.73 (0.05)	0.67 (0.03)	0.69 (0.03)				
- 11–18.7	8	0.64 (0.05)	0.63 (0.06)	0.75 (0.05)	0.74 (0.06)	0.66 (0.05)	0.68 (0.05)				
- 18.8–25.4	4	0.60 (0.05)	0.57 (0.06)	0.73 (0.05)	0.73 (0.03)	0.65 (0.07)	0.65 (0.05)				
- ≥25.5	6	0.65 (0.07)	0.56 (0.08)	0.74 (0.05)	0.71 (0.04)	0.66 (0.03)	0.68 (0.03)				
SF-36											
- MCS			* (0.1027)					0.981 / 0.31	0.8214	0.929 / 1.22	0.3120
- <50	31	0.64 (0.05)	0.59 (0.07)	0.74 (0.05)	0.73 (0.05)	0.66 (0.04)	0.68 (0.04)				
- ≥50	21	0.64 (0.04)	0.62 (0.04)	0.75 (0.04)	0.73 (0.04)	0.67 (0.02)	0.68 (0.03)				
- PCS					* (0.1449)	** (0.0377)	** (0.0173)	0.943 / 0.97	0.4136	0.878 / 2.21	0.0986
- <50	32	0.63 (0.05)	0.59 (0.06)	0.74 (0.04)	0.72 (0.05)	0.66 (0.04)	0.67 (0.04)				
- ≥50	20	0.65 (0.04)	0.61 (0.06)	0.75 (0.04)	0.74 (0.04)	0.67 (0.02)	0.70 (0.03)				
MIDAS		* (0.1720)						0.758 / 1.01	0.4373	0.790 / 0.85	0.5699
- 0–5	6	0.61 (0.03)	0.60 (0.09)	0.71 (0.05)	0.72 (0.02)	0.65 (0.03)	0.69 (0.05)				
- 6–10	7	0.65 (0.03)	0.59 (0.02)	0.74 (0.02)	0.73 (0.07)	0.67 (0.03)	0.66 (0.04)				
- 11–20	7	0.61 (0.05)	0.59 (0.05)	0.74 (0.05)	0.70 (0.06)	0.64 (0.03)	0.67 (0.03)				
- ≥21	17	0.65 (0.06)	0.59 (0.07)	0.76 (0.04)	0.74 (0.04)	0.67 (0.04)	0.69 (0.04)				

$\overline{x}(sd)$: Mean (standard deviation). *N* Frequency. ** Statistical significance *p* < 0.05. * 0.05 < *p-value* ≤ 0.20. *SF-36* Short Form 36 items, *MCS* Mental Component Scale, *PCS* Physical Component Scale

The multivariate analysis did not find significant differences in FA between ROIs of left or right sides according to the anxiety-depression levels or the scores of the quality of life or MIDAS questionnaires.

## Discussion

Our goal in this study was two-fold. First, to analyze if structural white matter abnormalities in pain-modulating regions are present in migraine, and whether they are associated with migraine frequency and therefore with a poorer prognosis. Second, to analyze if white matter changes are present in the aforementioned areas in individuals with higher cognitive reserve that would result advantageous in terms of migraine chronification. To this end, we performed a baseline DT MRI and a cognitive reserve assessment in a group of migraine patients and followed them up for 6 months.

Our results showed a significantly lower FA in several white matter tracts involving the anterior insula, cingulate gyrus, and uncinate fasciculus in those patients who still met the criteria for chronic migraine after 6 months of follow-up. The analysis at 3 months did not show significant differences, perhaps suggesting that ultrastructural white matter changes require time to develop. In this regard, migraine duration could have influenced the results but was not considered in this study.

We found a significant association between FA values in the selected ROIs mainly on the right side, and migraine frequency (*p* = 0.0276), that is, the higher number of headache days the less FA in the selected ROIs. Some authors did not find a correlation between connectivity and migraine frequency [19].

A multivariate analysis ruled out the role of different confounders in our results, including gender, use of preventive therapy, and migraine comorbidities such as anxiety or depression.

A significant influence of preventive drugs is unlikely given that the analysis for this variable showed no statistically significant between-group differences. Nonetheless, those patients on preventive therapy showed lower FA values in the left ROIs, mainly in the left insula and uncinate fasciculus. Since the left insula is often assigned to positive, parasimpathetically-dominated responses [33], it could be speculated that lower FA values in this region could be related to the presence of more prominent negative feelings in these patients.

The physical component score of the quality of life questionnaire was directly associated with the FA values in the uncinate fasciculus bilaterally, perhaps underscoring the role of this tract in anxiety [34]. Although depression is frequently reported as an important migraine comorbidity [35], we did not find an association between FA and this trait. In this study anxiety was associated with reduced FA in the right anterior insula, although the association was not confirmed by multivariate analysis. According to some authors, mood swings in migraine may result from altered right insula processing [18].

The ROIs analyzed in this study were selected for their known participation in pain processing, perception and modulation. Pain perception is a complex process involving pain-facilitating and pain-inhibiting brain regions [13]. Migraine involves numerous aspects of the pain experience, including affective (pain tolerance, self-awareness, fear, anxiety), sensory-discriminative, and cognitive domains (attention, expectation, pain memory). Migraneurs have lower interictal pain thresholds than controls, suggestive of abnormal sensory-discriminative processing, suggestive of abnormal affective response to pain [36]. The pain matrix can be divided into two pathway: the lateral (encoding location, intensity and quality of sensations) and the medial system, mediating the unpleasant-affective dimension of pain, the main components of this pathway , insula anterior, limbic connections an cingulate cortex, are the ROIs we targeted for this study [37].

The insula is functionally connected with the anterior cingulate gyrus [38] and both structures participate in affective pain processing. The insular cortex is implicated in both anticipatory and stimulus-related processing [39]. The anterior insula is involved in the awareness of the unpleasant feeling associated with pain, and the cingulate gyrus is involved in the marshaling of response to this unpleasantness [40]. When in pain, anterior insula activation is associated to pain relief. Sensory and cortico-limbic pathways converge on the anterior cingulate cortices contributing to varying degrees of cognitive evaluation to pain affect [41]. Regarding the uncinate fasciculus, it represents a bidirectional white matter tract connecting the lateral orbitofrontal cortex with the anterior temporal lobes and amygdala [42], and is part of the limbic system. Abnormalities in the uncinate fasciculus have been associated with memory, language and social emotional-processing problems but mainly with anxiety [34] and pain [43].

The structures studied here are implicated in pain modulation, emotional, affective and cognitive response to pain, and their dysfunction is consistent with the lower threshold and tolerance to pain with negative effects on mood and cognition displayed by chronic migraine patients [36]. The presence of DTI changes at baseline in those patients with the poorest prognosis (LTCM) may suggest that these abnormalities could predispose to chronic pain, as mentioned by Mansour et al. who found baseline white matter abnormalities in mesial prefrontal regions in patients who subsequently developed chronic lumbar pain [44]. Other authors hypothesize that repeated migraine attacks could induce used-dependent plastic changes in the white matter of selected regions related to pain modulation [20]. Whether atypical functional connectivity or white matter structural abnormalities might predispose individuals to migraine or are the result of recurrent migraines is still a matter of debate and research. Connectivity studies using resting state f-MRI in migraine have found an aberrant functional organization in pain modulating regions, and a positive relationship between migraine attack frequency or years with migraine, and the extent of atypical functional connectivity [36, 45].

These neurocognitive networks processing the perception of pain are modifiable negatively by repeated pain in patients with migraine [37], but can also be modified in the opposite direction, i.e., better tolerance to pain. In fact, increased FA values in the insula have been described in yoga practitioners, consistent with a strengthened insular integration of nociceptive input and parasympathetic autonomic regulation. Yogis, as opposed to controls, used cognitive strategies involving parasympathetic activation and interoceptive awareness to tolerate pain [46].

Our evaluation of cognitive reserve showed that FA values in the right ROIs (anterior insula, cingulate gyrus, and uncinate fasciculus) were significantly associated with CR (*p* = 0.0268) as shown by the multivariate analysis. Most of the networks involved in CR are served by a set of inter-related cognitive processes (arousal, sustained attention, response to novelty, and awareness) with a strongly right-hemisphere, fronto-parietal, representation [8]. Supporting the idea of the right anterior insula and cingulate gyrus intervening in effortful –related brain activation is the recent work of Engstron et al. [47], where they show that the right anterior insula is active during painful stimuli and pain expectation. In this sense, it has been demonstrated that the width of activation of the right anterior insula differentiates high and low resilient individuals. Resilient people are more flexible and appropriately adjust the level of emotional resources to meet the demand of a situation, in this case pain [14]. The fact that participants with higher CR had increased FA precisely in the right anterior insula and both cingulate gyri might suggest that a higher efficiency of these tracts would result in more powerful pain modulating networks.

CR is another potentially modifiable factor that may positively influence cerebral networks, contributing to content the anatomical and functional alterations present in chronic headache patients. The impact of differences in gray matter volume and white matter thickness on cognition is tempered by lifetime exposure to years of formal education, literacy level, occupational status, and engagement in leisure activities, consistent with the predictions of the cognitive reserve model, and thus is potentially modifiable by supplying appropriate experiences [7].

## Conclusion

In conclusion, longstanding chronic migraneurs show evidence of structural abnormalities in white matter pathways involving the anterior insula, cingulate and uncinated gyri, all of them structures implicated in pain modulation, emotion and perception. Changes are more pronounced on the right side, and may contribute to pain chronification. These tracts, particularly on the right side, are also implicated in cognitive reserve, which in turn seems to play an important role in pain conditions. CR potentiation could result in strengthening these tracts and their ability to positively influence chronic headache.

## Abbreviations

ROI: region of interest
CR: cognitive reserve
DTI: diffsusion-tensor imaging
DW: diffusion-weighted
LTCM: long-term chronic migraneurs
TFCE: threshold-free cluster enhancement
FA: fractional anisotropy
TBSS: Tract-based spatial statistics
CM: chronic migraine
