# Erratum to: Right fronto-insular white matter tracts link cognitive reserve and pain in migraine patients

**DOI:** 10.1186/s10194-016-0617-x

**Published:** 2016-03-11

**Authors:** Marian Gomez-Beldarrain, Isabel Oroz, Begoña Garcia Zapirain, Begoña Fernandez Ruanova, Yolanda Garcia-Chimeno, Alberto Cabrera, Ane Anton-Ladislao, Urko Aguirre-Larracoechea, Juan Carlos Garcıa-Monco

**Affiliations:** Service of Neurology Hospital de Galdakao-Usansolo, Galdakao, 48960 Vizcaya, Spain; DeustoTech-LIFE/eVIDA, Universidad de Deusto, Vizcaya, Spain; Research and Innovation Department, Magnetic Resonance Imaging Unit, OSATEK, Vizcaya, Spain; REDISSEC, Health Services Research on Chronic Patients Network Research Unit Hospital de Galdakao Usansolo, Galdakao, Vizcaya, Spain

After the publication of the original article [1] it was brought to our attention that one of the authors’ names was incorrectly reported by the authors. The original spelling reads as ‘Yolanda Garcia Fernandez’, however, the correct spelling of the name should read as Yolanda Garcia-Chimeno. Please accept the authors’ most sincere apologies for this mistake.
